# Preventive Effect of Salicylate and Pyridoxamine on Diabetic Nephropathy

**DOI:** 10.1155/2016/1786789

**Published:** 2016-11-30

**Authors:** Tarek Kamal Abouzed, Seiichi Munesue, Ai Harashima, Yusuke Masuo, Yukio Kato, Khaled Khailo, Hiroshi Yamamoto, Yasuhiko Yamamoto

**Affiliations:** ^1^Department of Biochemistry and Molecular Vascular Biology, Kanazawa University Graduate School of Medical Sciences, 13-1 Takara-machi, Kanazawa 920-8640, Japan; ^2^Department of Biochemistry, Faculty of Veterinary Medicine, Kafrelsheikh University, Kafr El Sheikh 33516, Egypt; ^3^Molecular Pharmacotherapeutics, Faculty of Pharmacy, Kanazawa University, Kakuma-machi, Kanazawa 920-1192, Japan

## Abstract

*Objective*. Diabetic nephropathy is a life-threatening complication in patients with long-standing diabetes. Hemodynamic, inflammatory, and metabolic factors are considered as developmental factors for diabetic nephropathy. In this study, we evaluated whether pharmacological interventions with salicylate, compared to pyridoxamine, could prevent diabetic nephropathy in mice.* Methods*. Male mice overexpressing inducible nitric oxide synthase in pancreatic *β*-cells were employed as a diabetic model. Salicylate (3 g/kg diet) or pyridoxamine (1 g/L drinking water; ~200 mg/kg/day) was given for 16 weeks to assess the development of diabetic nephropathy. Treatment with long-acting insulin (Levemir 2 units/kg twice a day) was used as a control.* Results*. Although higher blood glucose levels were not significantly affected by pyridoxamine, early to late stage indices of nephropathy were attenuated, including kidney enlargement, albuminuria, and increased serum creatinine, glomerulosclerosis, and inflammatory and profibrotic gene expressions. Salicylate showed beneficial effects on diabetic nephropathy similar to those of pyridoxamine, which include lowering blood glucose levels and inhibiting macrophage infiltration into the kidneys. Attenuation of macrophage infiltration into the kidneys and upregulation of antiglycating enzyme* glyoxalase 1* gene expression were found only in the salicylate treatment group.* Conclusions*. Treatment with salicylate and pyridoxamine could prevent the development of diabetic nephropathy in mice and, therefore, would be a potentially useful therapeutic strategy against kidney problems in patients with diabetes.

## 1. Introduction

Diabetic nephropathy is a life-threatening complication that occurs in 20–40% of patients suffering from diabetes and is the leading cause of chronic kidney disease [1]. There are many factors influencing the development of diabetic nephropathy, including genetic, environmental, hemodynamic, and metabolic factors [2]. Development of diabetic nephropathy is characterized by glomerular hyperfiltration, increased excretion of urinary albumin, and thickening of glomerular basement membranes followed by an expansion of the extracellular matrix in the mesangial areas. It ultimately progresses to glomerular sclerosis associated with renal dysfunction [3].

Hyperglycemia* per se* is a major cause of the onset and progression of diabetic complications. High glucose concentrations lead to vascular derangements through various molecular mechanisms including glycation, polyol pathway, hexosamine pathway, oxidative stress, and activation of protein kinase C [4]. These factors interact with one another, thereby facilitating host responses, including inflammatory reactions and stress responses, leading to glomerulosclerosis under diabetic conditions. Of them, our group and others focus on the glycation reaction and advanced glycation end-products (AGE) implicated in the pathogenesis of diabetic nephropathy [5, 6]. The receptor for AGE (RAGE) association with ligands triggers the acceleration of this disease [7]. For example, RAGE-overexpressing diabetic mice showed progressive glomerulosclerosis with renal dysfunction, compared to diabetic littermates lacking the RAGE transgene [8]. Moreover, homozygous RAGE-null diabetic mice failed to develop mesangial matrix expansion or glomerulosclerosis [9]. Intracellular RAGE signaling involves activation of transcriptional factor NF*κ*B activity, thus enhancing proinflammatory reactions [10, 11].

To prevent and treat diabetic kidney disease, we examined the effects of pyridoxamine, an antiglycating drug, and salicylate, an anti-inflammatory drug, on the development of diabetic nephropathy using our unique animal model. Pyridoxamine is one of the natural forms of vitamin B6 and is known to act as a nucleophilic trap for reactive carbonyl intermediates in AGE formation [12]. Pyridoxamine is reported to prevent the development of early stage nephropathy in a rat model of diabetes [13, 14]. The effectiveness on late stage diabetic nephropathy including glomerulosclerosis is not yet fully elucidated in a rat model of diabetes [13]. Salicylate is a well-known anti-inflammatory compound that inhibits I*κ*B kinase-*β* (IKK*β*) and is downstream of NF*κ*B [15]. Recent clinical studies have shown that salsalate, a nonacetylated salicylate, lowered fasting blood glucose levels and HbA1c as well as markers of inflammation [16, 17] but did not decrease the formation late glycation products [18].

In this study, we studied the effects of salicylate, compared to pyridoxamine, on early to late stages of diabetic nephropathy in our original mouse model of diabetic kidney injuries with insulin treatment group as a control.

## 2. Materials and Methods

### 2.1. Animals

The male mice of a type 1 diabetic mouse line that overexpresses inducible NO synthase (iNOS) in pancreatic *β*-cells and control CD1 (ICR), CD-1 background (Charles River, Japan), were used in this study [8, 19]. They were fed a high-calorie diet (Labo H standard; Nosan, Yokohma, Japan) that contained 31.1% protein, 8.2% lipids, 3.3% fiber, 6.3% ash, and 8.1% water. The transgenic mice consistently develop overt diabetes as early as 1 week after birth due to the NO-mediated selective destruction of insulin producing pancreatic *β*-cells, which results in advanced diabetic nephropathy [8, 19, 20]. Animals were treated in accordance with the Fundamental Guidelines for Proper Conducts of Animal Experiment and Related Activities in Academic Research Institutions under the jurisdiction of the Ministry of Education, Culture, Sports, Science, and Technology of Japan. Animal experiments were approved by the Committee on Animal Experimentation of Kanazawa University.

### 2.2. Animal Groups with Different Treatment

Pyridoxamine-dihydrochloride-monohydrate (4-aminomethyl-3-hydroxy-2-methyl-5-oxymethylpyridinedihydrochloride) was purchased from Tokyo Chemical Industry Co., Japan. The pyridoxamine dihydrochloride was given at 1 g/L (~200 mg/kg/day) in the drinking water from 4 to 20 weeks of age for 16 weeks, resulting in the serum concentration at 0.42 *μ*M (<0.03 *μ*M in nontreated mice). Salicylate (sodium salicylate, Sigma-Aldrich, Japan) was given at 3 g/kg in their diets, yielding the serum concentration at 102.6 *μ*M (not detected in nontreated mice). For the insulin treatment group, a long-acting insulin analog of insulin detemir (Levemir, Novo Nordisk) was subcutaneously injected twice a day at the dose of 2 units/kg body weight.

The following five animal groups were subjected to phenotypic analyses: (1) nondiabetic control (*n* = 8), (2) diabetic mice without treatment (*n* = 19), (3) diabetic mice with insulin treatment (*n* = 8), (4) diabetic mice with salicylate treatment (*n* = 12), and (5) diabetic mice with pyridoxamine treatment (*n* = 15).

### 2.3. Biochemical Assays

Urinary samples were collected by manual manipulation then kept in Eppendorf tubes at −80°C. Blood glucose levels were determined using Glucocard (Arkray, Japan). Body weight (BW) and nonfasting blood glucose levels were checked at 4, 8, 12, 16, and 20 weeks of age. Serum and urinary creatinine levels were monitored at 12, 16, and 20 weeks of age. The urinary albumin-creatinine ratio (ACR) was calculated at 20 weeks of age. Serum and urinary creatinine were measured using the creatinine-test (Wako, Osaka, Japan). Urinary albumin and beta-2 microglobulin levels were measured by a mouse Albumin ELISA Quantitation kit (AlbuWell II M, Exocell, Philadelphia, PA) and a mouse BMG/*β*2-MG ELISA kit (Elabscience, Japan), respectively.

### 2.4. Histopathology

After sacrificing the animals at 20 weeks of age, the left kidney was obtained and fixed in a Carnoy's solution (10% acetic acid, 60% methanol, and 3% chloroform) or 4% paraformaldehyde. For 48 hr fixation, paraformaldehyde and Carnoy's solutions were replaced by phosphate buffer saline (PBS) and 70% methanol, respectively. The kidney specimens were sectioned at 1 *μ*m thicknesses, followed by the periodic acid-Schiff (PAS), periodic acid-methenamine silver (PAM), or hematoxylin and eosin (H&E) stain and imaged using a light microscope (ZEISS microscope with Fujifilm digital camera HC 300Z). Quantitative examinations were done on at least 50 glomeruli per mouse. The severity of the renal sclerosis was scored by multiple analysts on an arbitrary scale from 0 to 4 [8, 9]. The mean glomerular area was determined as described previously [8, 9].

### 2.5. Flow Cytometry (FACS)

To evaluate macrophage infiltration, cells from the entire right kidney from each animal group at 20 weeks of age were subjected to FACS analysis. The kidney was cut into small pieces by scissors and digested by incubating with collagenase II (1 *μ*g/mL). Cells were then washed and resuspended in staining buffer (PBS with 2% FCS) containing FcBlock (BD Biosciences). Cells were stained by incubating for 15 min at 4°C in the dark with the following antibodies: CD45-PE (eBioscience), CD11b–APC-Cy7 (BD Biosciences), and F4/80-PE-Cy7 (BD Biosciences). Cells were resuspended in 200 *μ*L of staining buffer containing 0.2 *μ*g/mL propidium iodide (Sigma-Aldrich), filtered through a 100 *μ*m mesh, and analyzed by FACSAria II (BD Biosciences). To quantify viable cell numbers, liquid counting beads were employed from the BD Cell Viability kit (BD Biosciences). Data were transferred and analyzed with FlowJo software (Tree Star).

### 2.6. RNA Extraction and Real-Time Polymerase Chain Reaction (PCR)

Expression of mRNA was measured by quantitative real-time polymerase chain reaction (qRT-PCR). Kidney tissues were snap-frozen in liquid nitrogen and preserved at −80°C prior to RNA extraction. Total RNA was extracted using Trizol reagent and purified by RNeasy plus kit (QIAGEN) according to the manufacturer's instructions. Complementary DNA was synthesized by using Oligo (dT) primer AffinityScript QPCR cDNA Synthesis Kit (Agilent Technologies, USA) according to the manufacturer's instructions. The SYBR green qRT-PCR was performed with specific DNA primers (Table 1). Amplification and real-time fluorescence detection were performed using a model Mx3005P Real-Time QPCR system (Stratagene Products Division, Agilent Technologies) and the following protocol: an initial denaturation and polymerase activation step for 2 min at 95°C followed by 40 cycles at 95°C for 5 s and 60°C for 20 s. The mRNA expression levels were normalized to TATA-binding protein (*Tbp*).

### 2.7. Determination of Salicylate and Pyridoxamine Concentrations in Mouse Sera

Serum samples (40 *μ*L) were mixed with 5 *μ*L of 2 *μ*M cimetidine (internal standard), 5 *μ*L of water, and 150 *μ*L of 200 *μ*M acetonitrile, followed by centrifugation at 22,000 ×g for 10 min. A 3 *μ*L aliquot of supernatant was injected into LC-MS/MS system, consisting of a Nexera HPLC system (Shimadzu, Kyoto, Japan) coupled to a triple quadrupole mass spectrometer (LCMS-8040, Shimadzu). Chromatography for salicylate was performed by means of gradient elution (flow rate, 0.4 mL/min) as follows: 0–1.0 minute, 99% A/1% B; 1.0–4.0 minutes, 99% A/1% B to 5% A/95% B; 4.0–5.0 minutes, 5% A/95% B; 5.0–5.1 minutes, 5% A/95% B to 99% A/1% B; 5.1–7.0 minutes, 99% A/1% B [A, water containing 0.1% formic acid; B, methanol containing 0.1% formic acid] using ACQUITY UPLC BEH C_18_ (2.1 × 100 mm, 1.7 *μ*m; Waters, Milford, MA) at 50°C. Chromatography for pyridoxamine was performed by means of gradient elution (flow rate, 0.4 mL/min) as follows: 0–1.0 minute, 5% A/95% B; 1.0–6.0 minutes, 5% A/95% B to 50% A/50% B; 6.0–6.5 minutes, 50% A/50% B to 65% A/35% B; 6.5–7.5 minutes, 65% A/35% B; 7.5–7.7 minutes, 65% A/35% B to 5% A/95% B; 7.7–14.5 minutes, 5% A/95% B [A, water containing 0.1% formic acid; B, acetonitrile containing 0.1% formic acid] using SeQuant ZIC-cHILIC column (2.1 × 150 mm, 3 *μ*m; Merck Millipore, Darmstadt, Germany) at 50°C. The multiple reaction monitoring was set at 169.1 to 134.1 for pyridoxamine, 137.1 to 93.0 for salicylate, and 253.1 to 159.1 for cimetidine. Data analyses were carried out using LabSolutions (ver. 5.80, Shimadzu) and quantified using sample peak height.

### 2.8. Statistical Analysis

*P* values were calculated using two-tailed Student's* t*-test for pairwise comparisons and one-way analysis of variance (ANOVA), followed by Bonferroni's test for multiple comparisons, unless otherwise stated. A *P* value of <0.05 was considered statistically significant. Data are expressed as mean ± SEM.

## 3. Results

### 3.1. Diabetic Conditions

**Figure 1:**
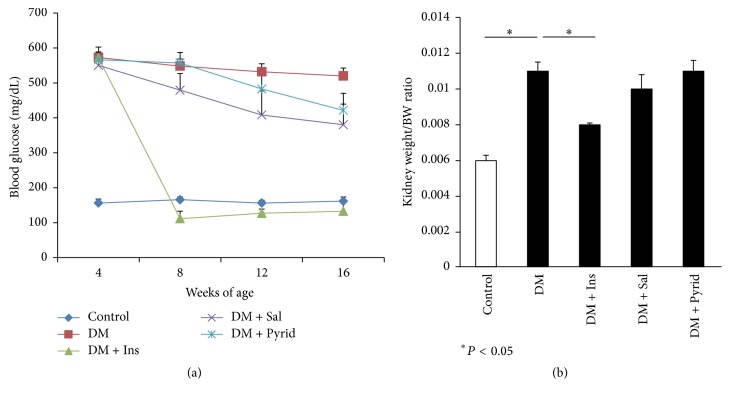
Blood glucose levels and nephromegaly. Nonfasting blood glucose levels in nondiabetic control (Control), diabetic mice without treatment (DM), diabetic mice with insulin treatment (subcutaneous injection of Levemir 2 units/kg body weight twice a day) (DM + Ins), diabetic mice with salicylate treatment (sodium salicylate 3 g/kg in the diets) (DM + Sal), and diabetic mice with pyridoxamine treatment (pyridoxamine dihydrochloride 1 g/L in the drinking water) (DM + Pyrid) (a). Kidney weight per body weight ratio (g/g) (b). Control (*n* = 8), DM (*n* = 19), DM + Ins (*n* = 8), DM + Sal (*n* = 12), and DM + Pyrid (*n* = 15). Values are mean ± SEM.

The nonfasting blood glucose levels were 500~600 mg/dL in diabetic mice at 4 to 16 weeks of age (Figure 1(a)), representing a sustained hyperglycemia. Insulin treatment twice a day significantly reduced hyperglycemia after the injection of the long-acting insulin analog (Figure 1(a)). Serious hypoglycemic troubles, including the death of the mice, were not seen in this study. Salicylate and pyridoxamine treatments in diabetic mice did not result in statistically significant decreases in nonfasting blood glucose levels, but there was a tendency of lowering the glucose levels in the salicylate treatment group (Figure 1(a)). The body weight (BW) of the diabetic groups was significantly lower than that of nondiabetic control, but no statistically significant differences were observed among diabetic groups with or without treatment at any time points during the observation period (Table 2 and data not shown). Food intake was significantly increased in diabetic mice when compared to nondiabetic controls (Table 2). Insulin treatment negated the increased food intake in diabetic mice; salicylate treatment also had a partial inhibitory effect on feeding behavior (Table 2). Pyridoxamine did not result in changes in food intake (Table 2). The same trends were noted in water drinking during the observation periods: nondiabetic control, 5.94 ± 0.281 mL/mouse/day; diabetes without treatment, 19.68 ± 0.454 mL/mouse/day; diabetes with insulin treatment, 4.26 ± 0.090 mL/mouse/day; diabetes with salicylate treatment, 13.35 ± 0.370 mL/mouse/day; diabetes with pyridoxamine treatment, 17.2 ± 0.776 mL/mouse/day.

### 3.2. Kidney Function and Histological Findings

**Figure 2:**
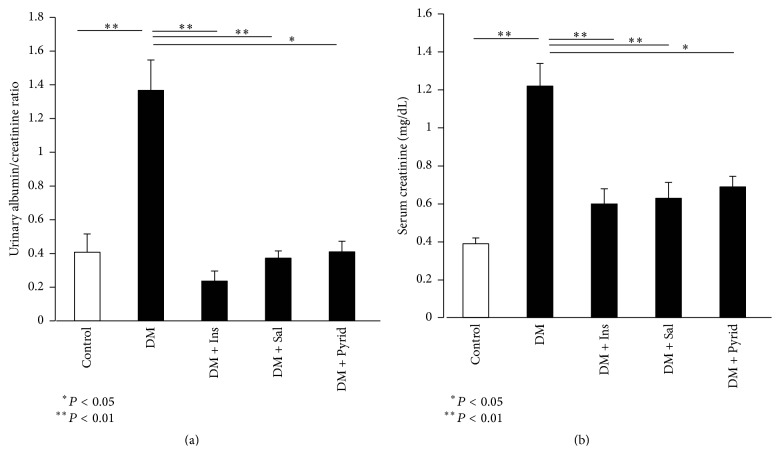
Albuminuria and serum creatinine levels. Albuminuria was evaluated as urinary albumin-creatinine ratio (ACR) in nondiabetic control (Control), diabetic mice without treatment (DM), diabetic mice with insulin treatment (DM + Ins), diabetic mice with salicylate treatment (DM + Sal), and diabetic mice with pyridoxamine treatment (DM + Pyrid) (a). Serum creatinine levels (mg/dL) (b). Control (*n* = 8), DM (*n* = 19), DM + Ins (*n* = 8), DM + Sal (*n* = 12), and DM + Pyrid (*n* = 15). Values are mean ± SEM.

After 16 weeks of treatment, functional and macro- and microscopic analyses of the kidneys were performed. The enlargement of the kidney, expressed as kidney weight per BW, was noted in diabetic mice (Figure 1(b)). Insulin treatment significantly ameliorated this renal size change, but treatment with salicylate or pyridoxamine did not (Figure 1(b)). In contrast, heart weight was not so different among all groups (Table 2). Albuminuria was assessed by the urinary albumin-creatinine ratio (ACR) in the animals at 20 weeks of age. Diabetic mice without treatment showed significant elevation of the ACR when compared to nondiabetic control mice (Figure 2(a)). All the treatment groups given insulin, salicylate, or pyridoxamine were found to significantly decrease the albuminuria seen in diabetic mice (Figure 2(a)). In this study, salicylate (3 g/kg diet) did not induce tubular damages such as high excretion of urinary beta-2 microglobulin. We next assessed serum creatinine levels in the mice which reflect filtration rate by the kidneys. The mouse serum creatinine levels in diabetes without treatment were significantly higher than those in nondiabetic controls at 16 weeks of age (Figure 2(b)). Treatment with insulin, salicylate, or pyridoxamine significantly attenuated the increased serum creatinine levels seen in diabetic mice (Figure 2(b)).

**Figure 3:**
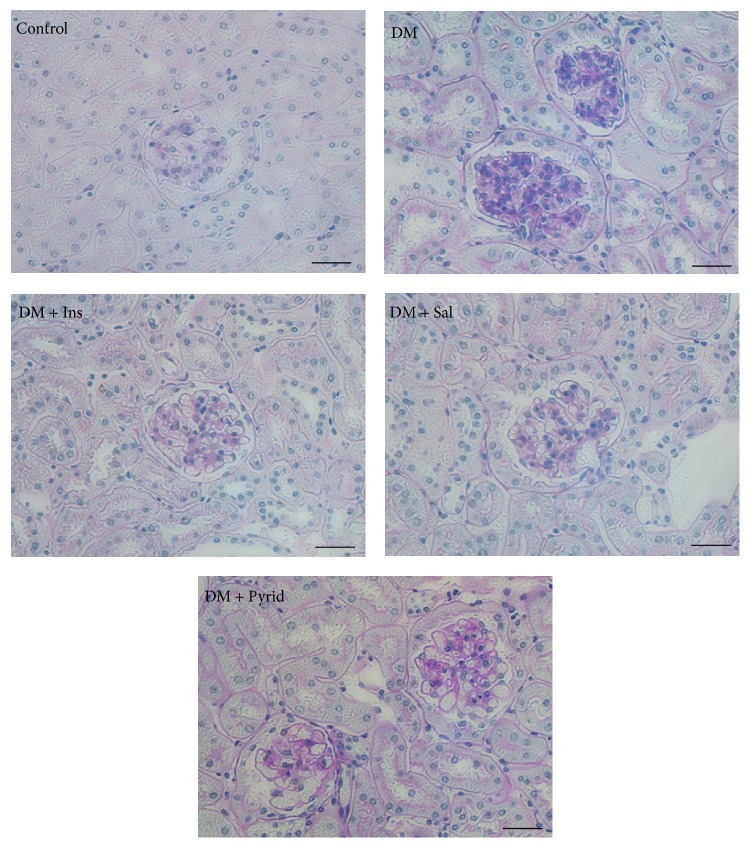
Periodic acid-Schiff (PAS) stain of kidneys. Nondiabetic control (Control), diabetic mice without treatment (DM), diabetic mice with insulin treatment (DM + Ins), diabetic mice with salicylate treatment (DM + Sal), and diabetic mice with pyridoxamine treatment (DM + Pyrid). Bar indicates 50 *μ*m.

**Figure 4:**
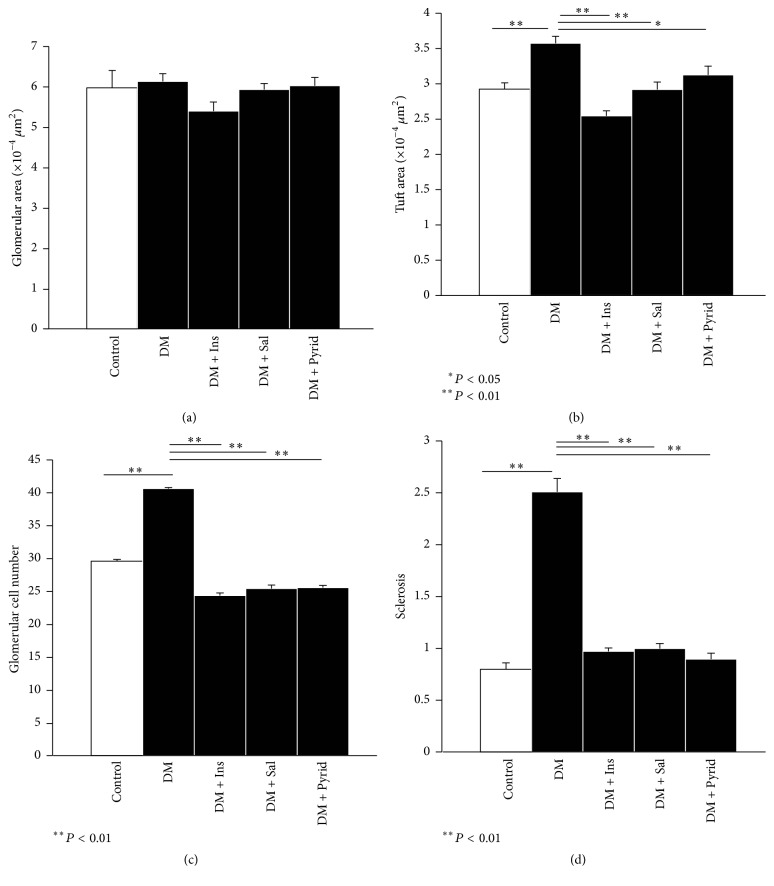
Histological evaluations of kidneys. Glomerular area (a), tuft area (b), glomerular cell number (c), and sclerosis indices (d) are shown. Nondiabetic control (Control) (*n* = 8), diabetic mice without treatment (DM) (*n* = 19), diabetic mice with insulin treatment (DM + Ins) (*n* = 8), diabetic mice with salicylate treatment (DM + Sal) (*n* = 12), and diabetic mice with pyridoxamine treatment (DM + Pyrid) (*n* = 15). Values are mean ± SEM.

Next, histopathological analyses were performed. The periodic acid-Schiff (PAS) staining of the kidney tissue sections is shown in Figure 3. Accumulation of PAS-positive materials in the glomeruli was prominent in the kidney from diabetic mice without treatment, when compared to nondiabetic controls. Either insulin, salicylate, or pyridoxamine treatment attenuated the deposition of the PAS-positive materials in the glomeruli (Figure 3). Quantitative evaluation of the histological indices was then performed. There were no significant differences in glomerular area among the groups (Figure 4(a)). However, the tuft area of the diabetic group without treatment was significantly larger than that of nondiabetic controls and the increased tuft area was significantly attenuated by the treatment with insulin, salicylate, or pyridoxamine in diabetic mice (Figure 4(b)). Glomerular cell number was counted and the glomerular cell number per tuft area was determined. This index was found to be increased under the diabetic condition (Figure 4(c)) and this upregulation was prevented by the treatment with insulin, salicylate, or pyridoxamine (Figure 4(c)). Glomerulosclerosis at 20 weeks of age was evaluated and expressed as sclerosis index. Sclerosis increased under diabetic conditions and was significantly prevented by the treatment with insulin, salicylate, or pyridoxamine (Figure 4(d)).

### 3.3. Macrophage Infiltration in Kidneys

**Figure 5:**
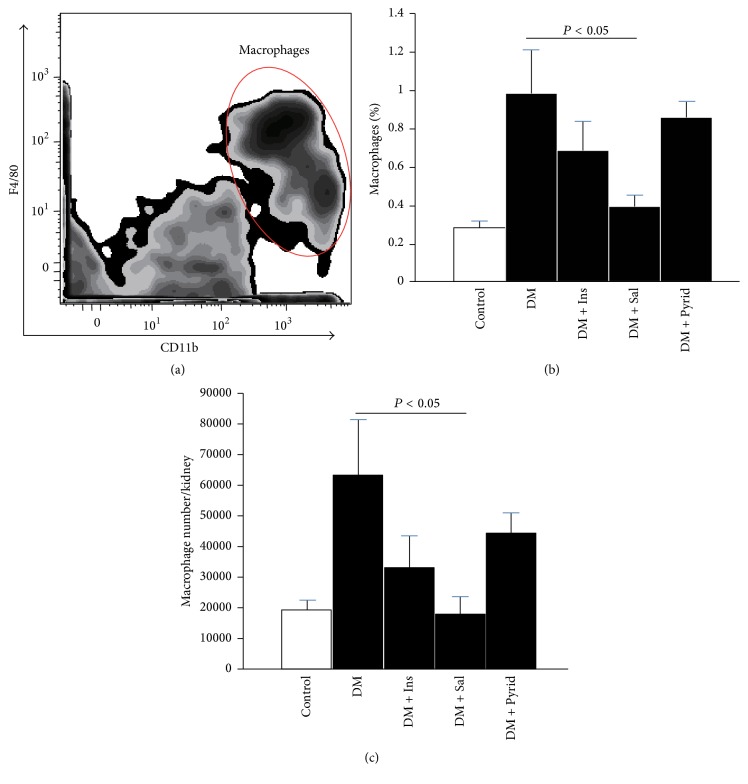
Flow cytometric data of kidneys. Macrophages were defined as PI^−^CD45^+^CD11b^+^F4/80^+^ cells in the kidney. The final gating panel of CD11b- and F4/80-positive cell cluster is shown (a). Macrophage % in the kidney (b) and the total number of the macrophages (c) were calculated. Nondiabetic control (Control) (*n* = 8), diabetic mice without treatment (DM) (*n* = 19), diabetic mice with insulin treatment (DM + Ins) (*n* = 8), diabetic mice with salicylate treatment (DM + Sal) (*n* = 12), and diabetic mice with pyridoxamine treatment (DM + Pyrid) (*n* = 15). Values are mean ± SEM.

We next focused on macrophage infiltration and accumulation evaluated by flow cytometry. Macrophages were defined as PI^−^CD45^+^CD11b^+^F4/80^+^ cells (Figure 5(a)). Although total living cell number in the whole kidney was invariant among each group (data not shown), macrophage % in the kidney and the total number of the macrophages were significantly increased under the diabetic condition and the increase was significantly blocked by the treatment with salicylate (Figures 5(b) and 5(c)). The tendency of downregulation in macrophage accumulation was observed in the groups with insulin or pyridoxamine treatment (Figures 5(b) and 5(c)).

### 3.4. Gene Expression Analyses of Kidneys

**Figure 6:**
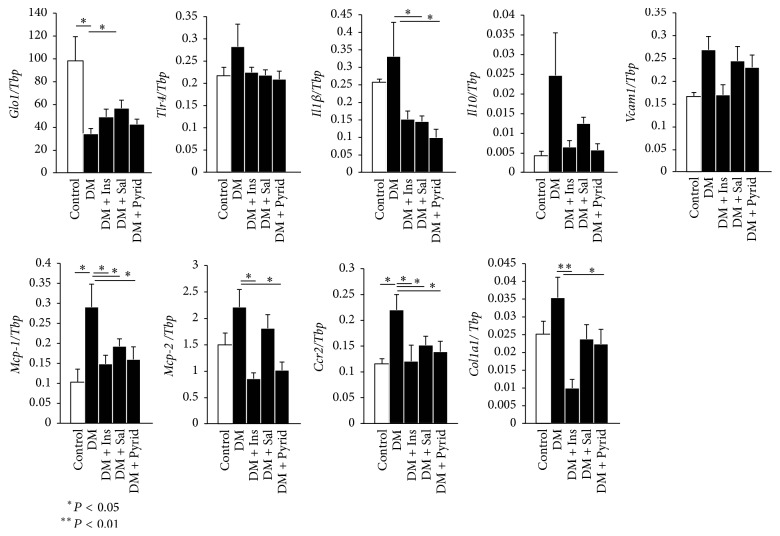
Gene expressions of kidneys. The mRNA expression levels were measured by qRT-PCR and normalized to TATA-binding protein (*Tbp*). Following primer sets were used for the detection (Table 1): glyoxalase 1 (*Glo1*), toll-like receptor 4 (*Tlr4*), interleukin (*Il*)*1b*,* Il10*, vascular cell adhesion molecule- (*Vcam-*)* 1*, monocyte chemoattractant protein- (*MCP-) 1*,* Mcp-2*, C-C chemokine receptor type 2 (*Ccr2*), and collagen 1a1 (*Col1a1*). Nondiabetic control (Control) (*n* = 8), diabetic mice without treatment (DM) (*n* = 19), diabetic mice with insulin treatment (DM + Ins) (*n* = 8), diabetic mice with salicylate treatment (DM + Sal) (*n* = 12), and diabetic mice with pyridoxamine treatment (DM + Pyrid) (*n* = 15). Values are mean ± SEM.

Inflammatory and fibrotic gene expressions were next examined. Expression of glyoxalase 1 (*Glo1*) mRNA which catalyze cytotoxic methylglyoxal, a highly active glycating agent, was significantly downregulated in kidneys from diabetic mice (Figure 6). Only salicylate treatment slightly, but significantly, upregulated* Glo1* mRNA (Figure 6). Toll-like receptor 4 (*Tlr4*) gene expression was not altered among the groups (Figure 6). Gene expression patterns were similar for interleukin (*Il*)*1β*,* Il10*, vascular cell adhesion molecule- (*Vcam-*)* 1*, monocyte chemoattractant protein- (*Mcp-*) 1,* Mcp-2*, C-C chemokine receptor type 2 (*Ccr2*), and collagen 1a1 (*Col1a1*) (Figure 6). The diabetic condition unregulated these mRNA levels in comparison with the nondiabetic sit and salicylate and pyridoxamine treatments downregulated the increase that was seen in the hyperglycemic condition (Figure 6).

## 4. Discussion

In this study, we have found preventive effects of salicylate and pyridoxamine on early stage indices of diabetic nephropathy, including albuminuria and enlarged tuft area, as well as late stage kidney injuries, such as an increase in serum creatinine and glomerulosclerosis. Salicylate was found to inhibit macrophage infiltration into the kidneys and to downregulate gene expressions of* Il1β*,* Mcp-1*,* Ccr2*, and* Col1a1*, showing anti-inflammatory and antifibrotic actions against diabetic kidney injury. Also, salicylate could significantly upregulate the antiglycating enzyme* Glo1* mRNA levels. The anti-inflammatory gene* Il10* was upregulated due to compensation in diabetic mice and downregulated after treatment with pyridoxamine or salicylate. On the other hand, pyridoxamine did not have a significant inhibitory action on macrophage infiltration but demonstrated downregulation of inflammatory and fibrotic gene expressions, such as* Il1β*,* Mcp-1*,* Mcp-2*,* Ccr2*, and* Col1a1*. In the present study, we isolated total RNAs from mouse whole kidneys for the analyses of various gene expressions. However, because the extracted RNAs and the resultant data were derived from more than 90% of tubular/interstitial components, it is likely that the present findings may be limited and confounding for interpreting glomerular changes. Further histological examinations may help to understand the expressions of these molecules in specific cell types of the kidneys.

Salicylates are among the most commonly used nonsteroidal anti-inflammatory drugs and nonacetylated (e.g., sodium salicylate, salsalate, and trilisate) and acetylated (aspirin) forms of salicylate are widely used to reduce fever, pain, and inflammation that occur with conditions such as rheumatic arthritis. Even at low doses, aspirin effectively inhibits cyclooxygenase enzymes COX 1 and 2 through covalent transacetylation of active site serine residues [21]. Nonacetylated salicylates are reported not to modify activities of cyclooxygenase enzymes [22]. At a higher concentration, both nonacetylated salicylates and aspirin are known to inhibit the IKK*β*/NF-*κ*B axis, an important regulator of inflammation [23, 24]. Aspirin is reported to inhibit glycoxidation and AGE-cross-link formation [25] but not glycation itself. In a clinical study, treatment with salsalate, a nonacetylated salicylate, resulted in noninhibitory effects on AGE formation [18]. In this study, we used a high dose of sodium salicylate at 3 g/kg diet, resulting in the serum concentration at 102.6 ± 4.6 *μ*M, corresponding to the dose for human therapy, and could see inhibition of the development of diabetic nephropathy in mice by nonacetylated sodium salicylate. A previous study demonstrates that obesity activates the IKK*β*/NF-*κ*B pathway in animals and that inhibition of this pathway by salicylates improves obesity-induced diabetes [22, 26]. In addition, clinical studies show that salicylates halve the NF-*κ*B activity in circulating immune cells [27]. An animal study showed that salicylates induced a 50% decrease in the number of Ly6C^hi^ monocytes which express CCR2 mediating the migration of monocytes into local inflammatory sites [28]. This is compatible with our findings in this study. Moreover, it is interesting that salicylate induced the gene expression of* Glo1*, which catalyzes the conversion of cytotoxic methylglyoxal to S-D-lactoylglutathione and D-lactate, which suppresses glycation-mediated cellular damage associated with diabetes and aging.

Pyridoxamine, a vitamin B6, is reported to have many effects including (1) inhibition of AGE formation by trapping dicarbonyl intermediates during glycation reaction, (2) scavenging toxic carbonyl products of glucose and lipid degradation, and (3) trapping of reactive oxygen species (ROS) [12, 29]. The dosage of pyridoxamine (~200 mg/kg/day) used in this study, achieving the serum concentrations at 0.42 ± 0.29 *μ*M, was within a less toxic range and its preclinical efficacy has been proven in other animal models of early diabetic nephropathy, such as KK-Ay/Ta and streptozotocin-induced diabetic rats [13, 14]. The serum concentration of pyridoxamine was lower than our expectations and previous reports [30]; this may be due to its instability in aqueous solutions and photosensitivity as well as different administration methods. Using our mouse model of diabetic nephropathy, pyridoxamine treatment with 200 mg/kg/day significantly improved early to late stages of kidney injuries.

In conclusion, this study demonstrated that an anti-inflammatory reagent salicylate as well as an antiglycooxidative drug pyridoxamine could inhibit the development of diabetic nephropathy in mice. Both drugs significantly blocked diabetes-induced inflammatory and profibrotic gene expressions. Attenuation of macrophage infiltration into the kidneys and upregulation of* Glo1* mRNA expression were found only in the salicylate treatment group. Synergistic and additive effects of the combination with salicylate and pyridoxamine as well as therapeutic effects of these drugs will hopefully be investigated in future studies. Interventional strategies using these drugs may be useful for diabetic nephropathy.

## Figures and Tables

**Table 1:** DNA primer pairs used in this study.

	Forward primer	Reverse primer
*Il-1β*	5′-GCT CAG GGT CAC AAG AAA CC -3′	5′- CAT CAA AGC AAT GTG CTG GT-3′
*Il-10 *	5′- TGT TTC CAT TGG GGA CAC TT-3′	5′- AAC TGG CCA CAG TTT TCA GG-3′
*Tbp*	5′- ACC CTT CAC CAA TGA CTC CTA TG-3′	5′-TGA CTG CAG CAA ATC GCT TGG-3′
*Tol4 *	5′- GGG TCA AGG AAC AGA AGC AG-3′	5′- GCT CAT TTC TCA CCC AGT CC-3′
*Mcp-1*	5′- TCC CAA TGA GTA GGC TGG AG-3′	5′- AAG TGC TTG AGG TGG TTG TG-3′
*Col1a1 *	5′- GCT CCT CTT AGG GGC CAC T-3′	5′- ATT GGG GAC CCT TAG GCC AT-3′
*Glo1 *	5′- TTG GTC ACA TTG GGA TTG CC-3′	5′- TCC TTT CAT TTT CCC GTC ATC AG-3′
*Mcp-2*	5′- GAA GAT GGT TAT CGT CAC CAC C-3′	5′- CGT TCC AGG CAT TGT ACC ACT-3′
*Ccr2 *	5′- ATC CAC GGC ATA CTA TCA ACA TC-3′	5′- TCG TAG TCA TAC GGT GTG GTG-3′
*Vcam1 *	5′- TTG GGA GCC TCA ACG GTA CT-3′	5′- GCA ATC GTT TTG TAT TCA GGG GA -3′

**Table 2:** Diabetic parameters in 16-week-old diabetic mice with or without treatment.

	Control	DM	DM + Ins	DM + Sal	DM + Pyrid
BW (g)	57.7 ± 1.5	46.2 ± 1.1^*∗*^	41.0 ± 1.5^*∗*^	40.4 ± 1.0^*∗*^	40.1 ± 0.9^*∗*^
Food intake (g/d)	4.58 ± 0.08	6.80 ± 0.06^*∗*^	3.95 ± 0.03	5.69 ± 0.13^*∗*^	6.43 ± 0.12^*∗*^
Heart (g)	0.195 ± 0.005	0.204 ± 0.007	0.191 ± 0.010	0.182 ± 0.004	0.184 ± 0.005

^*∗*^ *P* < 0.05 versus Control; data are expressed as mean ± SEM.
